# Experimental transmission of ovine atypical scrapie to cattle

**DOI:** 10.1186/s13567-023-01224-3

**Published:** 2023-10-20

**Authors:** Timm Konold, John Spiropoulos, Janet Hills, Hasina Abdul, Saira Cawthraw, Laura Phelan, Amy McKenna, Lauren Read, Sara Canoyra, Alba Marín-Moreno, Juan María Torres

**Affiliations:** 1 Department of Pathology and Animal Sciences, Animal & Plant Health Agency Weybridge, Addlestone, UK; 2 Central Unit for Sequencing and PCR, Animal & Plant Health Agency Weybridge, Addlestone, UK; 3 Centro de Investigación en Sanidad Animal (CISA-INIA-CSIC), Valdeolmos, Madrid, Spain

**Keywords:** Transmissible spongiform encephalopathy, prion, atypical scrapie, bovine, PMCA, mouse bioassay, veterinary neurology

## Abstract

Classical bovine spongiform encephalopathy (BSE) in cattle was caused by the recycling and feeding of meat and bone meal contaminated with a transmissible spongiform encephalopathy (TSE) agent but its origin remains unknown. This study aimed to determine whether atypical scrapie could cause disease in cattle and to compare it with other known TSEs in cattle. Two groups of calves (five and two) were intracerebrally inoculated with atypical scrapie brain homogenate from two sheep with atypical scrapie. Controls were five calves intracerebrally inoculated with saline solution and one non-inoculated animal. Cattle were clinically monitored until clinical end-stage or at least 96 months post-inoculation (mpi). After euthanasia, tissues were collected for TSE diagnosis and potential transgenic mouse bioassay. One animal was culled with BSE-like clinical signs at 48 mpi. The other cattle either developed intercurrent diseases leading to cull or remained clinical unremarkable at study endpoint, including control cattle. None of the animals tested positive for TSEs by Western immunoblot and immunohistochemistry. Bioassay of brain samples from the clinical suspect in Ov-Tg338 and Bov-Tg110 mice was also negative. By contrast, protein misfolding cyclic amplification detected prions in the examined brains from atypical scrapie-challenged cattle, which had a classical BSE-like phenotype. This study demonstrates for the first time that a TSE agent with BSE-like properties can be amplified in cattle inoculated with atypical scrapie brain homogenate.

**Supplementary Information:**

The online version contains supplementary material available at 10.1186/s13567-023-01224-3.

## Introduction

Bovine spongiform encephalopathy (BSE), first described as a neurological disease in cattle in 1987 [1], was caused by the feeding of cattle with meat and bone meal (MBM) contaminated with a transmissible spongiform encephalopathy (TSE) agent that led to an epidemic affecting more than 180 000 cattle in the United Kingdom (UK) alone and is responsible for currently 178 cases of variant Creutzfeldt-Jakob disease in humans in the UK [2]. One of the measures introduced to monitor the epidemic was enhanced surveillance for TSEs, not only in cattle but also in sheep and goats, which were known to be affected by a TSE called scrapie. This resulted in a discovery of new types of TSEs in these species, such as atypical BSE (H- and L-type) in cattle [3, 4] and atypical scrapie in sheep and goats [5], that may have been undetected in these species in the past, and—unlike the now termed classical types of BSE and scrapie—may be sporadic in older animals.

Transmission studies of naturally occurring TSE agents in ruminants aimed to determine whether the species are susceptible, whether the disease can be diagnosed by the current post-mortem TSE tests and whether they resemble or can be distinguished from classical BSE. It is still unknown which TSE agent was recycled in MBM and hypotheses include a scrapie agent or a cattle-specific TSE agent [6]. Classical scrapie produced a disease in cattle by the intracerebral route, which was unlike classical BSE [7–10]. Transmission studies of atypical BSE to mice and hamsters suggested that the agent could develop classical BSE-like properties on subsequent passages [11–14] and thus be the original prion responsible for the BSE epidemic although this has neither been replicated in sheep [15] nor in cattle [16] even after repeated passages. More recently, it was suggested that the atypical scrapie agent may be the agent recycled in MBM and the source of classical BSE, based on protein misfolding cyclic amplification (PMCA) and bioassays that detected a classical BSE agent in atypical scrapie sheep brains, bovine and ovine transgenic mice [17] and minipigs [18] inoculated with atypical scrapie brain isolates.

The current study reported here was initiated to assess susceptibility of cattle to the atypical scrapie agent using two different brain homogenates and to describe the clinical, pathological (immunohistochemistry), molecular (Western immunoblot) and biological (mouse bioassay) disease phenotype if transmission occurred. It also provided the opportunity to examine brains from inoculated cattle by PMCA to evaluate whether the findings described in other species could be replicated.

## Materials and methods

### Inocula

Brains from two different naturally occurring atypical scrapie cases, frozen at −80 °C and defrosted prior to preparation, were used as test inocula for cattle groups 1 and 2; physiological saline solution was used as control inoculum for group 1. Both brains were classified as atypical scrapie based on results of Western blot and immunohistochemistry.

Test group 1 inoculum was defrosted brainstem from a 5-year old Brecknock Hill Cheviot ewe (76/06) with prion protein gene (*PRNP*) genotype homozygous for A_136_F_141_R_154_Q_171_ as determined by *PRNP* sequencing [19], which was tested as fallen stock. This inoculum was also inoculated in transgenic mice as part of a separate project, which did not transmit to Bov-Tg110 mice on first passage but transmitted to Ov-Tg338 mice based on Western immunoblot (WB) and immunohistochemistry (IHC). Infectious titre in Ov-Tg338 mice by serial dilution of the inoculum was calculated as 10^8.73^ LD_50_/g tissue (J Spiropoulos, unpublished)—see below for mouse strains.

Test group 2 inoculum was defrosted whole brain from an adult Welsh Mountain ewe (1088/06) with *PRNP* genotype homozygous for A_136_H_154_Q_171_, which was also tested as fallen stock. Brain tissue from this sheep, which generated a lesion profile in Ov-Tg338 that was compatible with natural cases of atypical scrapie, was also used for a successful oral transmission study in sheep [20].

For inoculation of mice, rostral medulla and cerebellum were collected sterile from cattle at necropsy, frozen at −80 °C and defrosted prior to preparation.

All inocula were tested for microbial contamination; test group 1 and 2 inocula were treated with ampicillin (1.25 mg/12.5 µL per 1 mL inoculum) and gentamicin (0.25 mg/6.25 µL per 1 mL inoculum) prior to use. Brain homogenate was prepared as 10% homogenate in physiological saline solution and 1 mL and 20 µL each used for inoculation of cattle and mice respectively.

### Animals and procedures

All procedures involving live animals were approved by the Home Office under the Animal (Scientific Procedures) Act 1986 and only undertaken following review and approval by APHA’s Animal Welfare Ethical Review Body (project licence numbers 70/7745, P47CEB089, P1956057D).

For the large animal component, 13 cattle were used. All were Holstein-Friesian crossbred castrated male calves at 5–7 months of age, obtained from a farm in Great Britain. After a pre-inoculation examination to confirm cattle suitable to go on study cattle were inoculated intracerebrally under general anaesthesia: premedication with 1 mg/10 kg bodyweight (BW) xylazine (Rompun 2%, Elanco) and 10–20 mg/10 kg BW propofol (PropoFlo Plus, Zoetis) given intravenously (induction), maintenance with isoflurane (Isoflo, Zoetis, 3–5% in O_2_) via endotracheal tube, reversal with 0.25 mg/10 kg BW intravenous atipamezole (Antisedan, Vetoquinol). All animals were also given antibiotics (Amoxicillin, 150 mg/10 kg BW Amoxypen LA, MSD) and analgesics [5 mg/10 kg BW meloxicam subcutaneously (Metacam, Boehringer-Ingelheim); 0.6 mg buprenorphine intramuscularly (Vetergesic, Ceva)]. The inoculation procedure with site and depth of inoculation was performed as described previously [21]. Five calves in test group 1 were inoculated with AFRQ/AFRQ brain homogenate and another group of five were inoculated with saline solution to serve as age-matched controls, which were housed in a separate pen but same air space. A group size of five was considered sufficient to detect a 50% infection rate with 95% confidence. Test group 2 consisted of two calves which were inoculated with AHQ/AHQ brain homogenate; they were housed in a separate pen in the same building but together with a non-inoculated calf that served as companion. A group size of 2 would be able to detect an infection rate of 78% with 95% confidence and differed because of different funding streams.

Cattle were observed twice daily during normal husbandry procedures (cleaning, feeding). More detailed clinical monitoring consisted of quarterly clinical examinations from 6 months post-inoculation (mpi) [21], which included tests of over-reactivity to assess responses to external stimuli [stick test, flash test, clipboard test, bang test and hand clap [22], weekly passive observations from 12 mpi [21], and weekly observation of the rising behaviour using video surveillance from 23 (group 1) and 18 (group 2) mpi, which was scored from 0 (normal) to 3 (unable to get up) as described in a separate study [23]. Due to the unavailability of surveillance cameras in the saline-inoculated group, which were kept separate, their rising behaviour could only be scored once they were moved to a pen with cameras or mixed with other cattle. All cattle were also weighed monthly from 12 (group 1) and 7 (group 2) mpi. For the purpose of this study, clinical signs suggestive of a TSE that were assessed included nervousness/apprehension in the corridor, head shyness/restless behaviour when restrained in a crush, over-reactive to tactile stimuli (e.g. skin prick during cranial nerve assessment), unexpected startle (once/multiple), abnormal tests of over-reactivity (repeated over-reactivity in at least one test), abnormal gait (mild-equivocal/obvious incoordination), difficulty rising, response to scratching of the tail head (lip licking or head movements) [16], tremor, and weight loss.

Rodent bioassay was carried out in transgenic mice overexpressing the ovine VRQ *PRNP* allele on a murine *PRNP* null background (Ov-Tg338) [24] and mice overexpressing the bovine *PRNP* (Bov-Tg110) [25], which were generated in-house, using brain from steers. As the outcome was unknown, brain donors would be clinically affected steers with or without confirmed prion disease or randomly selected steers if no disease occurred. Groups of 8 male or female weaned mice at 6–20 weeks of age were anaesthetised in an anaesthetic chamber with 3.5–5% isoflurane (Isoflo, Zoetis) in O_2_. After subcutaneous administration of an analgesic [Carprofen, Rimadyl, Zoetis: 100 µL of Rimadyl (50 mg/mL) in 4.4 mL of sterilized water for injection; final concentration 1.1 mg/mL] they were inoculated intracerebrally using a 25G × 5/8-inch needle as described previously [26]. Mice were kept in individually ventilated cages in groups of 4 and clinically monitored weekly for signs of a TSE from 30 days post-inoculation using a scoring system of 1 (normal), 2 (at least one major sign associated with TSE, such as ataxia, paresis, tremor, vacant stare, kyphosis or lordosis), and + (marked display of a major sign).

### Prion protein genotyping

EDTA blood samples were taken for analysis of the bovine prion protein gene including the promoter region [PrP promoter 23 and 12 bp (bp) insertions and deletions (indels)] and full open reading frame (ORF): DNA was extracted and amplified by polymerase chain reaction to determine bovine ORF polymorphisms and compare it to a wild-type PrP gene reference sequence from a Jersey cow, GenBank accession number AJ298878 [27]. For bovine PrP promoter indels a TaqMan allelic discrimination assay was used. The methods have been described in more detail previously [28, 29].

### Post-mortem examination

Cattle with signs suggestive of a TSE at clinical end-stage (definite clinical signs [21]) or any other non-treatable disease that affected their welfare were euthanized with pentobarbitone (minimum 80 mg/kg, Pentoject 20%, Animalcare) administered intravenously after intramuscular injection with 0.05–0.3 mg/kg xylazine (Rompun 2%, Elanco). As it was unknown whether cattle would develop disease at all, study end-point was set to a minimum of 96 mpi. Euthanasia of an animal in test group 1 would result in the euthanasia of a saline-inoculated control. Unfortunately, the last of the group 1 controls (steer 01/20) had to be euthanized because of an intercurrent disease so that no age-matched control was available for the last remaining atypical scrapie-inoculated steer 02/21. Instead, the non-inoculated steer in group 1 was retained for longer to provide a control for both 02/21 and the steers in group 2. As part of the necropsy protocol a range of nervous and other peripheral tissues (except for one control steer which was euthanised at a weekend) were taken and one half fixed in buffered formalin or formal saline (central nervous system tissues) and the other half was used fresh or kept frozen at −80 °C for further use, except for one case with confirmed *Mycobacterium bovis* infection where fresh tissue was not utilised further. Fresh obex tissue was used to detect proteinase-resistant prion protein (PrP^res^) by rapid TSE test, BioRad TeSeE ELISA (BioRad Laboratories), carried out according to the manufacturer’s instructions and WB according to previously published protocols using monoclonal antibodies Sha31 (BioRad Laboratories, included in the WB kit, dilution as per WB kit protocol), which targets the epitope ^156^YEDRYYRE^163^, 12B2 (Central Veterinary Institute of Wageningen UR; dilution 0.5 µg/mL), which targets epitope ^101^WGQGG^105^ and SAF84 (Cayman Chemicals; dilution 0.4 µg/mL), which targets epitope ^175^RPVDQY^180^ of the bovine PrP [30]. Three antibodies were used because they target different domains of PrP^res^ and aid in differentiation of bovine BSE strains: Sha31 as core-specific, 12B2 as N-terminus-specific and SAF84 as C-terminus specific antibody [30, 31].

Examination of fixed brain sections (obex, rostral medulla, caudal and rostral midbrain, thalamus, cerebellum, occipital, parietal and frontal cortex) for disease-specific prion protein (PrP^Sc^) was carried out by IHC using monoclonal antibody R145 (APHA Weybridge) at a 1/150 dilution, which targets aa 221–232 of the bovine PrP sequence [32]. Nine brains sections were also examined for vacuolation (Haematoxylin-Eosin).

Mice were euthanized in a CO_2_ chamber (fill rate of 30–70% of the chamber volume per minute with CO_2_) if they scored + in three or the 1st and 3rd week of three consecutive weeks or showed any significant deterioration in health, which could also include non-TSE-related conditions. Collected tissues were brain and spleen (fixed/frozen); brain was sectioned at 4 different levels (frontal, thalamic, midbrain and medulla) and examined by IHC for PrP^Sc^ as described for cattle, using antibody R145 at a 1/1000 dilution.

### Protein misfolding cyclic amplification

PMCA was carried out whilst some of the animals were still alive, thus excluding two animals (one control, one atypical scrapie-inoculated steer of group 1) as well as the steer infected with *M. bovis*. The original sheep inoculum was also included in the examination.

The methodology for PMCA was as described previously [17]: 5 µL of the source inoculum, which was prepared in sterile saline solution, was suspended in 45 µL of substrate (tenfold dilution), and each test material was tested in triplicates; dextran supplementation (Sigma-Aldrich, 6500–10 000 Dalton) with 2.5 µL of dextran 5% in distilled water per PMCA reaction (dextran final concentration 0.25%); Zirconia bead (BioSpec) supplementation of 4 beads of 1 mm diameter per PMCA reaction; seven PMCA rounds in total, with one round lasting 24 h with 48 cycles of 20 s of sonication and 29 min + 40 s of incubation at 37 °C. Sonication was performed in a Q700 sonicator (Qsonica) at amplitude level 30. Rounds were performed by taking 5 µL of sonicated samples and diluting it in 45 µL of fresh substrate. Non-inoculated tubes were included as negative controls. The substrate was prepared as 10% weight-volume homogenate of Bov-Tg110 brains in PMCA buffer. The PMCA buffer consisted of 50 mM Tris-HCl pH 7.4, 5 mM EDTA, 300 mM NaCl and 1% Triton x-100. One pill of complete protease inhibitor was added to 50 mL of PMCA buffer prior to brain homogenization. Positive controls were included in the PMCA reactions to check for successful amplification: bovine BSE brain and primary passaged Bov-Tg110 murine brain inoculated with atypical scrapie that tested negative for PrP^res^ [17].

After each round PMCA reaction products were examined by WB for the presence of PrP^res^ as previously described [33–35] using monoclonal antibodies, Sha31 (1 µg/mL), which targets the epitope ^156^YEDRYYRE^163^ of the bovine PrP, and 12B2 (4 µg/mL), which targets epitope ^101^WGQGG^105^ of the bovine PrP sequence (see above). For the PrP^res^ digestion, two proteinase K (PK) treatment procedures were used, which differed in their PK concentration, incubation temperature and duration, and digestion buffer. PK treatment A consisted of a PK digestion protocol for atypical PrP^res^ detection as previously described [35], using 40 µg/mL of proteinase K in buffer 5% sarkosyl, 5% Triton X100, 1 M Urea and 16 mM Tris–HCl (pH 9.6) at 60 °C for 15 min. PK treatment B consisted of a PK digestion protocol for PMCA amplification products [33, 34] with higher PK concentration for a better resolution of the classical PrP^res^ profile and comparison with classical BSE. Under this protocol samples were incubated for 45 min at 37 °C using 200 µL of a 100 µg/mL of proteinase K solution in buffer A of the commercial TSE ELISA (TeSeE, Bio-Rad Laboratories). For PrP^Sc^ deglycosylation, N-Linked glycans were removed after PK treatment by using a peptide-*N*-glycosidase (PNGaseF+) F kit (New England Biolabs) according to the manufacturer’s instructions.

### Thermostability assay of PMCA positive samples

PrP^res^ positive samples after 7 rounds of PMCA amplification in Bov-Tg110 brain substrate were subjected to thermostability assay as previously described [36]. This assay is used to distinguish prion strains exposed to heat treatment, with classical BSE isolates retaining their infectivity and seeding activity in contrast to other scrapie strains (e.g. RML), which show a reduction in both parameters. Briefly, samples were placed in safe-lock tubes (Eppendorf), heated at 98 °C for 2 h in a thermocycler (Primus 96 Plus Thermal Cycler, MWG AG Biotech) and allowed to cool gradually to room temperature, then stored at − 20 °C and defrosted prior to PMCA. An additional PMCA round in Bov-Tg110 substrate was performed with heated and non-heated samples and results were checked by WB as described above.

## Results

Table 1 lists survival time, genotype and cause of death in the cattle of groups 1 and 2. Presence of clinical signs associated with BSE in cattle prior to cull is displayed in Table 2.

**Table 1:** **Cattle genotypes, survival times and cause of cull**

Group	Animal	Inoculum	ORF	23 bp	12 bp	Survival time [mpi]	Cause of cull
2	92/20	AHQ/AHQ brain	6:6 Q78 het	+/−	+/−	106	Timed cull
	54/20	AHQ/AHQ brain	6:6	−/−	−/−	106	Timed cull
	1/21	None	6:6	−/−	−/−	112	Downer cow (injury)
1	2/21	AFRQ/AFRQ brain	6:6 Q78 hom	+/−	+/−	106	Timed cull
	2/20	AFRQ/AFRQ brain	6:6 Q78 het N192 het	+/−	+/−	91	Tumour (soft tissue sarcoma)
	**6/17**	AFRQ/AFRQ brain	6:6 Q78 hom	+/+	+/+	48	**TSE suspect**
	6/16	AFRQ/AFRQ brain	6:6 Q78 hom	+/+	+/+	46	Bovine tuberculosis^a^
	4/15	AFRQ/AFRQ brain	6:6 Q78 hom	+/+	−/−	23	Hip fracture
	1/20	Saline	6:5	−/−	+/−	94	Downer cow (foot abscess)
	55/20	Saline	6:6 Q78 het	+/−	+/−	91	Timed cull
	17/17	Saline	6:6 Q78 het	+/−	+/−	51	Timed cull
	8/17	Saline	6:5	−/−	+/−	50	Timed cull
	14/15	Saline	6:6 Q78 hom	+/+	+/+	33	Timed cull

ORF: open reading frame of the bovine PrP gene detailing the number of N-terminal octapeptide repeats, the silent polymorphisms Q78 and N192, either homozygous (hom) or heterozygous (het) at position 78 and 192 of the ORF respectively compared to the wild type; and the 23 and 12 bp indels (− deletion allele, + insertion allele) of the promoter PrP gene, mpi: months post-inoculation or post-test group inoculation for 1/21, rounded down to the nearest month. The TSE suspect is highlighted in bold letters.

^a^This case has been reported separately [56].

**Table 2:** **Presence of clinical signs associated with BSE within 4 weeks prior to death**

Signs	92/20	54/20	1/21^a^	2/21	2/20	6/17	6/16^b^	4/15	1/20	55/20	17/17	8/17	14/15
	Atypical scrapie		Control	Atypical scrapie					Control				
Nervous/apprehensive in corridor	−	−	−	−	−	+	−	−	−	−	−	−	−
Head shy/restless in crush	−	−	−	−	−	+	−	+	−	−	−	−	−
Over-reactive to tactile stimuli	−	−	−	−	−	−	−	+	−	−	−	−	−
Unexpected startle	−	−	−	−	−	+	−	−	−	−	−	−	−
Abnormal tests of over-reactivity	−	−	−	−	−	+	−	−	−	−	−	−	−
Abnormal gait	−	−	−	−	−	−	−	−	−	−	−	−	−
Difficulty rising	−	−	+++	−	−	+	?	?	+++	?	?	?	?
Scratch test	−	−	−	−	−	?	−	−	−	−	−	−	−
Tremor	−	−	−	−	−	+	−	−	−	−	−	−	−
Weight loss	+	+	−	−	−	?	?	−	+	−	−	−	−

+ = present; − = absent; for difficulty rising scores: 0 = −, 1 = +, 2 = ++, 3 = +++, ? = not determined within 4 weeks of death.

^a^Last exam 39 days prior to death.

^b^Last exam 45 days prior to death.

Two cattle (both controls: 1/20—group 1, 1/21—group 2) became recumbent (rising score: 3). Steer 1/20 had a foot abscess diagnosed when lame, which improved following treatment, but was found recumbent and no alternative diagnosis was found to explain it. Steer 1/21 was knocked down by its pen mate and then unable to get up. Both had previously rising scores of 1. None of the atypical scrapie-inoculated cattle had a score exceeding 1; slight difficulty rising was either characterized by rocking several times before getting up (Additional file 1) or delayed rising of the hind limbs. Rising behavior scores in relation to times post-inoculation are displayed in Table 3.

**Table 3:** **Rising behavior scores of cattle**

Group	2			1				
Animal	92/20	54/20	01/21^a^	02/21	02/20	06/17	06/16	01/20^a^
First score of 1 (mpi)	82	27	29	60	50	48	Never	91
N score 1/N obs	3/87 (3%)	15/87 (17%)	41/93 (44%)	36/79 (46%)	2/65 (3%)	1/20 (5%)	0/19 (0%)	3/3 (100%)

N = number of.

^a^Control cattle; both 1/20 and 1/21 were downer cows (score 3) prior to death.

**Figure 1:**
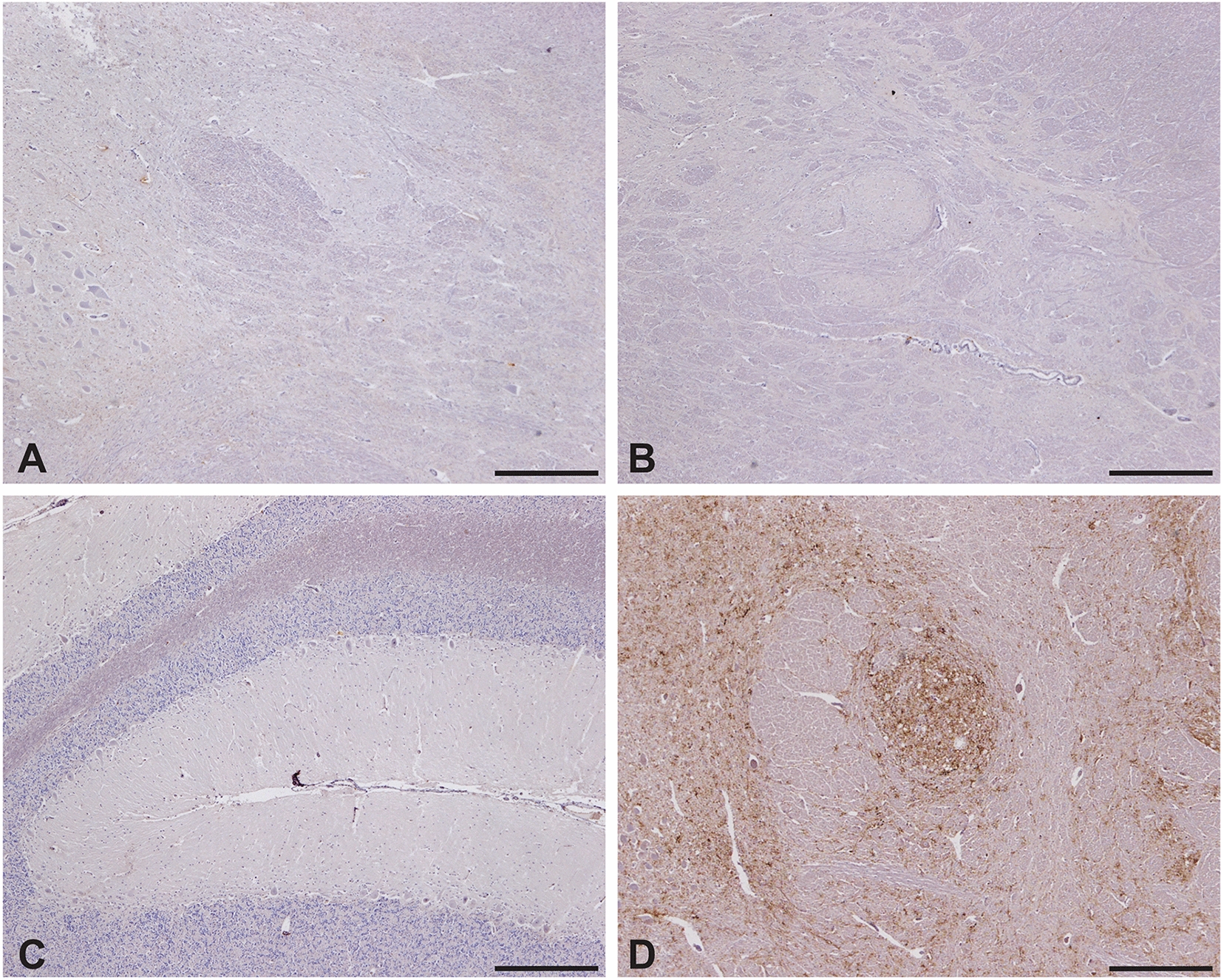
**Immunohistochemical examination of the brain of TSE suspect 06/17.** IHC of the nucleus of the solitary tract (**A**), spinal tract nucleus of the trigeminal nerve (**B**) and cerebellum (**C**) with no disease-specific immunolabelling. **D** demonstrates IHC of the nucleus of the solitary tract from a positive control, run alongside the other samples. R145 antibody, scale bar represents 500 mm.

**Figure 2:**
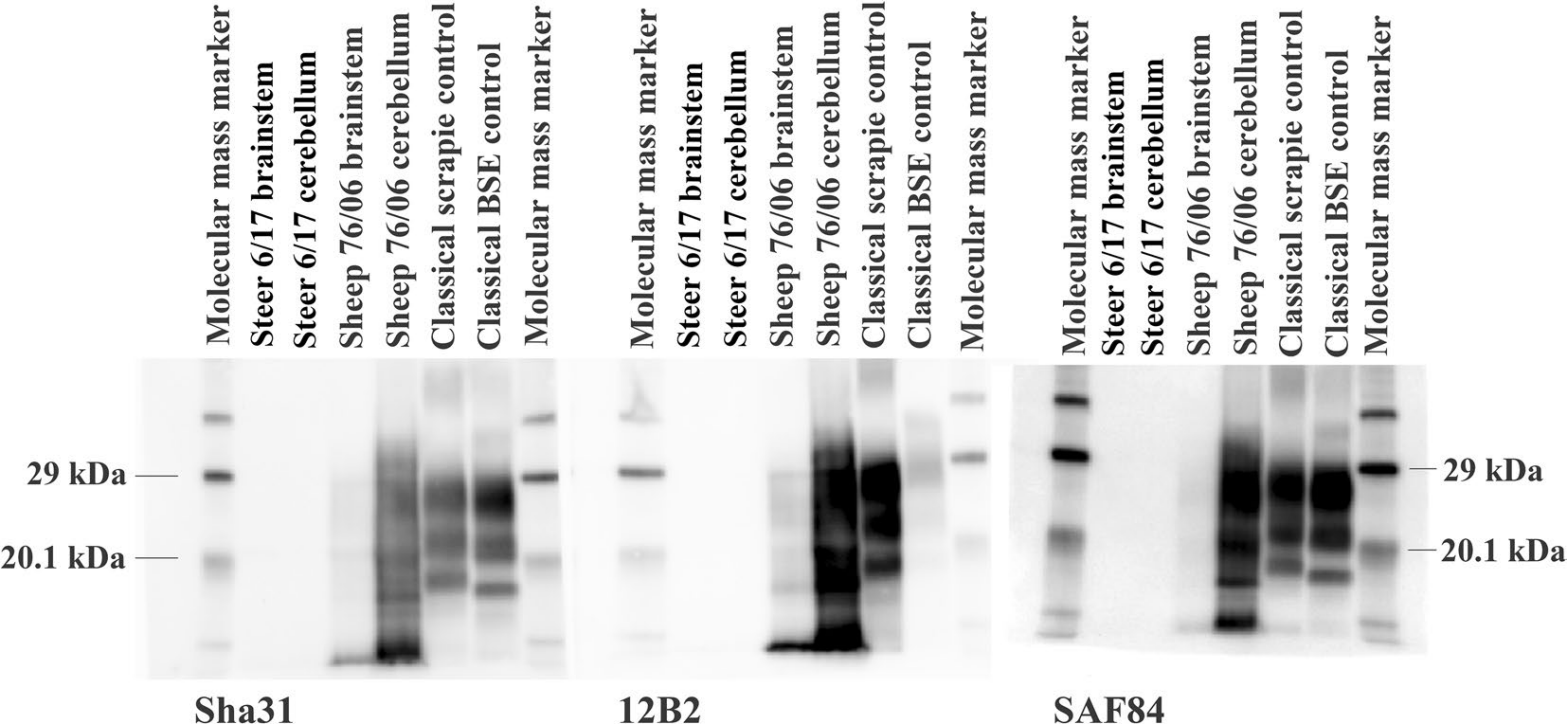
**Western immunoblot examination of the brainstem of TSE suspect 06/17.** Atypical scrapie sheep 76/06 was the inoculum donor of test group 1, which included clinical suspect 6/17. PrP^res^ was not detectable with any of the antibodies in the samples from steer 6/17.

None of the cattle had a prion disease confirmed by the rapid (ELISA) or confirmatory post-mortem TSE tests (WB, IHC) despite one steer, 6/17 of group 1, displaying suspected signs of a TSE at 45 mpi, characterized by increased agitation when restrained in the crush and hind limb muscle tremor, and progression of signs led to its euthanasia at 48 mpi. Figures 1 and 2 show the negative immunohistochemical result and WB result, respectively, from this steer, representative of the confirmatory TSE test results in all cattle. Histopathological examination of the brain from the clinically affected steer did not reveal any lesions suggestive of BSE or any other inflammatory condition; there was, however, white matter vacuolation restricted to the ventral area of the rostral midbrain. Clinical signs displayed by this steer were nervousness, head and rump muscle tremor, over-reactivity to external stimuli (forceful kicking when the hind legs were touched with a stick = positive stick test) and difficulty rising (rocking multiple times before getting up), see Table 2. The clinical signs are shown in more detail in a movie file (Additional file 1). At the time, the animal was moved to a neighboring building with 6/16, which was suspected of having bovine tuberculosis, and after cull of 6/16 remained in this building with a younger steer as companion.

There was no evidence of transmission of a prion disease when rostral medulla and cerebellum of TSE suspect 6/17 were inoculated in transgenic mice: neither TSE-specific vacuolation nor accumulation of disease-specific PrP accumulation were demonstrated by histopathology and immunohistochemistry although some were judged to display signs of a TSE (Table 4).

**Table 4:** **Survival times of transgenic mice inoculated with brain from TSE suspect 06/17**

Brain tissue	Mouse strain	Positive/N mice	Mean survival time [d] (SEM)	Clinically positive mice/N mice
Rostral midbrain	Ov-Tg338	0/8	487 (51)	5/8
	Bov-Tg110	0/7	445 (34)	4/7
Cerebellum	Ov-Tg338	0/8	622 (69)	1/8
	Bov-Tg110	0/6	468 (42)	3/6

Mice lost shortly after inoculation are excluded

*N* total number, *SEM* standard error of mean.

**Figure 3:**
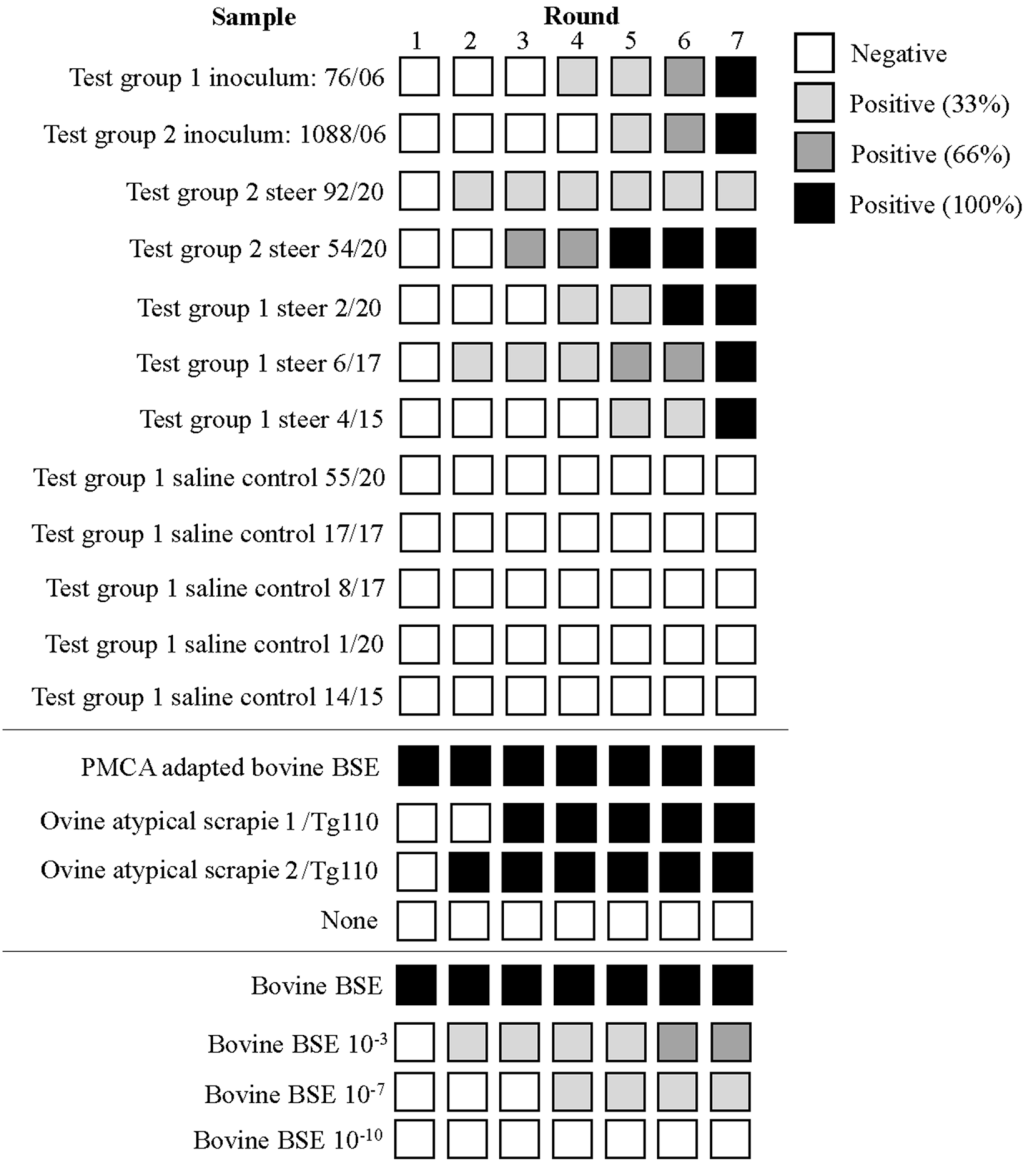
**Schematic representation of the PMCA results of cattle samples.** The results obtained for the 7 rounds of amplification for each inoculum are represented by colored squares. Darker color relates to higher number of positives tubes within triplicates. Positive controls: Cattle BSE inocula adapted to PMCA amplification in Bov-Tg110 brain (PMCA adapted bovine BSE), Bov-Tg110 mice inoculated with atypical scrapie but negative for PrP^res^ detection (ovine atypical scrapie 1/Tg110 and ovine atypical scrapie 2/Tg110; these controls were added to mimic the steer situation after challenge with atypical scrapie). Negative controls: non-inoculated Bov-Tg110 substrate (none). Bovine BSE PMCA amplification titration in Bov-Tg110 substrate (bovine BSE, bovine BSE 10^−3^, 10^−7^ and 10^−10^).

**Figure 4:**
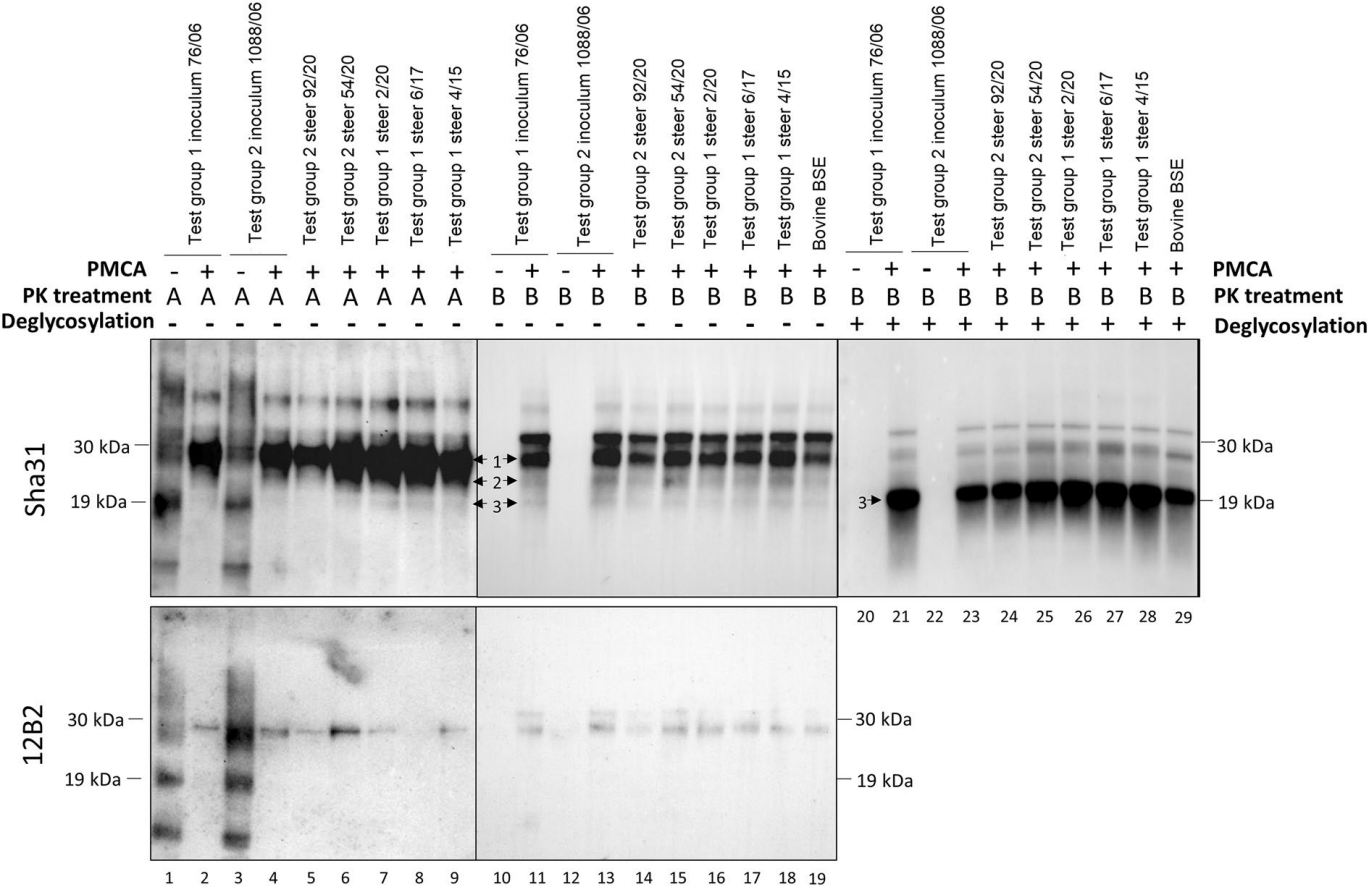
**Western immunoblot analysis of the PMCA products from cattle brains.** Western immunoblot PrP^res^ signature of the PMCA products. Treatment A (PK digestion protocol for atypical PrP^res^ digestion). PrP^res^ signature changes from atypical (lanes 1 and 3) to a classical 3 band pattern (lanes 2, 4–9) after PMCA amplification in Bov-Tg110 substrate. Treatment B (higher PK concentration for PMCA amplification). PrP^res^ signature of the PMCA amplification products in the Bov-Tg110 substrate (lanes 11, 13–19) is indistinguishable from the BSE profile after PMCA amplification (lane 10). Atypical PrP^res^ (lanes 10 and 12) was not detected by WB when using this digestion protocol. Deglycosylation treatment with PNGaseF. The non-glycosylated PrP^res^ of the atypical scrapie PMCA amplification products (lanes 21, 23–28) have a molecular mass of 19 kDa, identical to the non-glycosylated PrP^res^ BSE control (lane 29). Arrows point to PrP^res^ bands: (1) diglycosylated band, (2) monoglycosylated band and (3) non-glycosylated band. The bands observed above the diglycosylated band (arrow 1) may represent aggregates of PrP^res^ generated by PMCA, the intensity of which are reduced after the deglycosylation treatment. 12B2 1/4000, Sha31 1/5000. Molecular mass markers in kilodaltons (kDa) are indicated on the sides of the blots.

**Figure 5:**
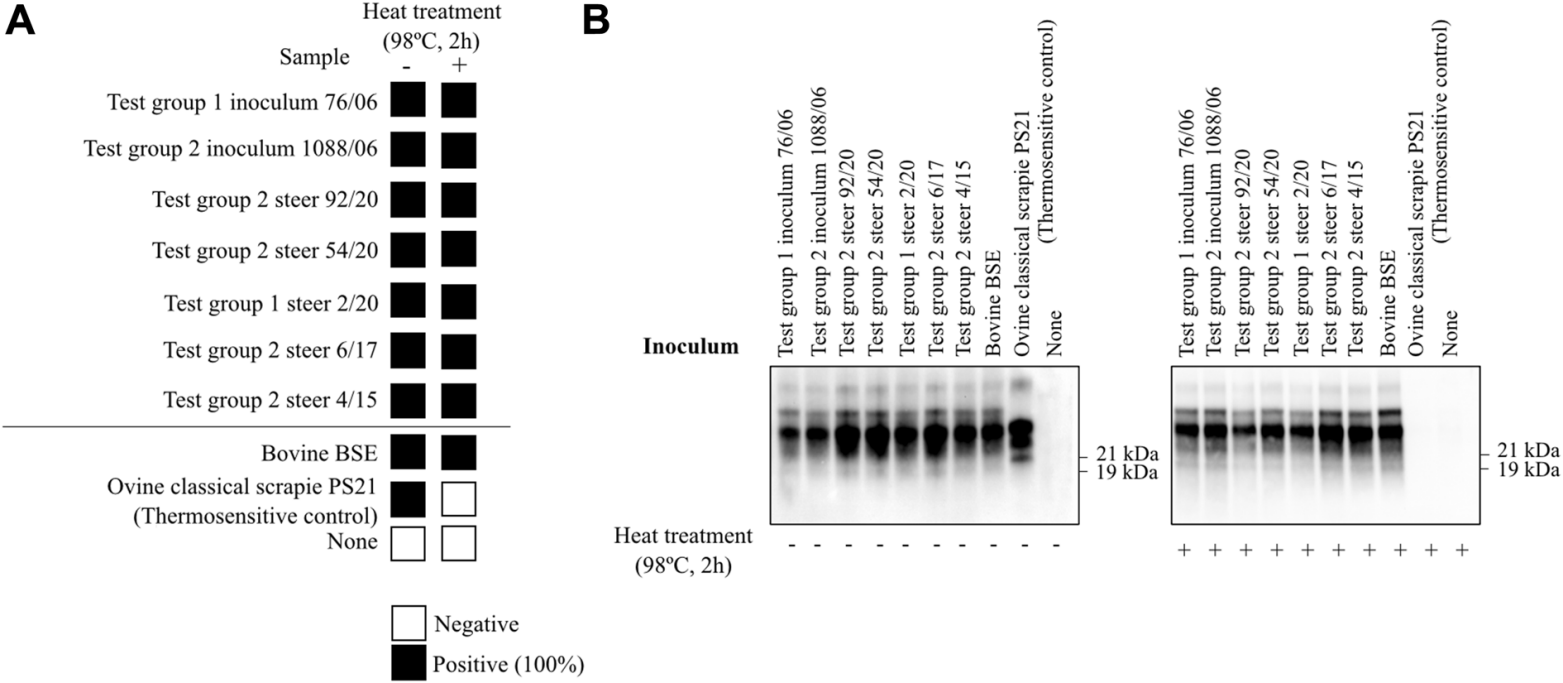
**Thermostability analysis of the positive PMCA products.** **A** (left) Schematic representation of the PMCA results after heat treatment. The results obtained for each inoculum are represented by colored squares. Darker color relates to positives tubes. Positive controls: Bovine BSE. Negative controls: classical scrapie thermosensitive control (PS21 thermosensitive control), non-inoculated Bov-Tg110 substrate (none). **B** (right) Western immunoblot analysis of the PMCA products after heat treatment. PrP^res^ signature is BSE after PMCA amplification in Bov-Tg110 substrate following heat treatment proving thermostability. Only the thermosensitive control failed to amplify. Sha31 1/5000. Molecular mass markers in kilodaltons (kDa) are indicated on the right side of the blots.

PMCA of the brain of all examined steers produced a positive WB result in those steers that were inoculated with atypical scrapie brain homogenate whereas the saline inoculated control cattle were all WB negative (see Figure 3). The molecular profile of the PMCA products from the positive reactions was undistinguishable from classical BSE (Figure 4). After PNGase treatment, PMCA products displayed a predominant non-glycosylated band of 19 kDa identical to the 19 kDa non-glycosylated band of classical BSE. Furthermore, heat treatment of PMCA positive samples at 98 °C and subsequent PMCA amplification in Bov-Tg110 substrate and WB analysis demonstrated that the samples were as thermoresistant as the control BSE sample (Figure 5).

## Discussion

Transmission studies by the intracerebral route in cattle using TSE isolates not originally found in cattle (classical scrapie [7–10], transmissible mink encephalopathy and chronic wasting disease [37]) produced disease with a survival time ranging from 14 to 54 months post-inoculation that was confirmed by post-mortem tests, although not all inoculated cattle developed clinical or pathologically confirmed disease on first passage [7, 9]. Clinical disease could be classified as two syndromes, a nervous and a dull form [9]. Despite small sample sizes in the present study, it was anticipated that some cattle would develop disease, and indeed one steer inoculated with brain homogenate from an AFRQ/AFRQ atypical scrapie sheep developed a neurological disease characterized by tremor, nervousness, over-reactivity to external stimuli and slight difficulty rising, the combination of which was highly suspicious of BSE, and the survival time of 48 months was within the range of transmission studies in cattle with different isolates. More than 60% of naturally infected cattle with classical BSE observed in different countries displayed nervous behavior; muscle tremor ranged from 33 to 82%, kicking during milking from 30 to 50%, and difficulty rising was observed in up to 33% of cattle, which will lead to recumbency in advanced cases. Although the majority of cattle display behavioral, sensory and locomotor changes simultaneously, some may only display signs in two of these categories [38]. A positive stick test was generally rare in experimentally infected BSE cases [39], which were used to being tested routinely, so its presence in this steer, even when simply confined to a crush without touching its legs, was even more suspicious of an alteration in sensation or behavior that was suggestive of BSE. At the time the animal was kept separate with another steer that developed tuberculosis, which may have been an additional stressor that triggered more severe signs [40]. Difficulty rising, which may have presented as rocking several times before getting up, brief dragging of hind legs or recumbency, was a consistent finding in all cattle with experimentally produced atypical BSE [16, 23]. However, it was seen at various degrees in most animals in the present study, including controls where video surveillance was available to check it, and may be the result of joint degeneration as the animals grow larger rather than TSE-related, particularly since it was usually mild (rocking several times to get up) and inconsistent. Unexpectedly, the post-mortem confirmatory tests were negative for a prion disease in the clinical suspect, and there was also no evidence of infectivity when brain from this animal was inoculated in transgenic mice, which are a sensitive bioassay model, at least on primary passage. A similar outcome is not unprecedented since it has been shown that cattle even when inoculated with BSE brain homogenate can display suspicious signs without BSE confirmation post-mortem [39]. In one study where a similar phenomenon was observed, further transmission studies in bovine transgenic mice were also negative. PMCA detected seeding activity at low levels, lower than for confirmed experimentally produced BSE cases, and it was hypothesized that breed rather than prion protein gene difference accounted for this difference [41]. As the cattle in the present study were not purebred, we cannot rule out that cross-breeding had an effect on the clinical presentation. Focal spongiosis of the white matter has been reported as pathological finding in unconfirmed clinical suspects but neither its etiology nor clinical significance is known, and it is found also in BSE cases [42]. It was considered an incidental finding in the clinically affected steer, and there was no evidence of hepatic dysfunction suggested as possible cause [43].

A considerable number of mice had signs suggestive of TSE, which led to their euthanasia, although there was no evidence of a TSE. Compared to cattle, a neurologic examination of mice is in general more difficult because of their small size and more focused on passive observations. In addition, a systematic examination protocol as it exists for cattle with TSEs [39, 44] does not exist for mice. It cannot be ruled out that some of the signs displayed were incorrectly interpreted or not specific enough (e.g. kyphosis, vacant stare, gait in older mice with possible joint disease that were interpreted as ataxia) but absence of detectable prion protein or vacuolation has also been reported in clinically affected mice inoculated with the BSE agent on first passage, even though it became detectable after serial passages [45]. Despite mice being supposedly kept throughout their natural life span, mean survival times were less than 650 days and generally shorter in Bov-Tg110 mice compared to Ov-Tg338 mice because of conditions outlined above. Taking into consideration the species barrier and based on a previous transmission study of atypical scrapie to Bov-Tg110 mice where survival times exceeded 500 days in positive mice even on second passage [17], it is possible that the survival times were too short to confirm transmission of a TSE. Mouse brains were not subjected to PMCA to assess whether they contained levels of PrP^Sc^ not detectable by IHC and further subpassages in mice are currently under consideration.

In general, the cause of clinical signs in prion diseases remains unknown and may not involve PrP^Sc^ [46], which would explain why the TSE suspect steer was not different to others in terms of post-mortem test results, although it would be concerning if similar instances occurred in natural disease because of the failure to diagnose the disease.

In the present study, only one steer developed signs suggestive of BSE, but all atypical scrapie-inoculated cattle tested by PMCA (five of seven) amplified prions but had a negative BSE confirmatory test. The level of seeding activity per round (number of positive tubes per round) did not appear to have any association with clinical disease because 100% of positive reactions were achieved in clinically unremarkable steer 54/20 in earlier rounds than for clinical suspect steer 6/17 (Figure 3). The numbers are too small to determine whether genetic differences account for the variability: all steers were Holstein-Friesian crosses and only one other steer shared the same PrP gene polymorphism as the BSE suspect but was culled 2 months earlier without signs of BSE.

A recent study has shown that PMCA can amplify prions from ovine atypical scrapie isolates that are indistinguishable from classical BSE in cattle, which led to the hypothesis that atypical scrapie may be the origin of the classical BSE epidemic [17]. However, the same isolates inoculated into transgenic mice expressing the bovine prion protein gene did not produce confirmed disease in most mice on first passage, but the number of TSE-positive mice increased after subsequent passages with a 100% attack rate with most isolates after the third passage. In the current study only primary transmission of the brain from the clinical suspect to Bov-Tg110 mice was performed because this steer was considered most likely to have a prion disease. These findings are in line with the results in cattle where none of the inoculated cattle developed a prion disease that was confirmed by conventional tests. However, PMCA amplified BSE-like prions in all examined cattle brains, corroborating the results obtained for a similar experiment with pig brains [18].

Previous work describing the emergence of classical BSE from atypical scrapie transmission in Bov-Tg110 mice proved the utility of PMCA in Bov-Tg110 substrate to detect such emergence as being 1500-fold more sensitive than bioassays in Bov-Tg110 [17]. Therefore, the brain material obtained after the atypical scrapie transmission to steers were subjected to the same PMCA approach in order to unravel classical BSE emergence, and a BSE-like profile was obtained. The Sha31 antibody is able to detect a PrP^res^ pattern characterized by predominance of the diglycosylated band while showing a non-glycosylated band of 19 kDa. In the same line of results, the 12B2 antibody is not able to detect any true PrP^res^ signal, a feature common in classical BSE-related prions. The low faint signals detected by the 12B2 antibody could represent either PK or a minor subpopulation of PrP^res^ molecules preserving the 12B2 epitope as has been already discussed in previous work [47]. A second passage of atypical scrapie in cattle would be needed to assess whether a BSE-like disease can be produced eventually. Inoculation of the brain from one steer in bovine transgenic mice did not produce a confirmed TSE, which may not be unexpected based on the previously mentioned study [17] because not all isolates produced disease in mice after the second passage and the mean survival time of Bov-Tg110 mice with less than 500 days may not be long enough to confirm disease. Further subpassages in mice would be required to address this question.

This is the first study in cattle inoculated with naturally occurring scrapie isolates that found the presence of prions resembling classical BSE in bovine brain although this was limited to detection by the ultrasensitive PMCA. The results from thermostability assay confirmed that the isolates were as thermoresistant as the BSE agent as proven in other studies [36, 48]. Previous PMCA studies with various British atypical scrapie isolates did not find any evidence of amplification [49, 50]. This may be explained by the use of ovine brain as substrate rather than brain from Bov-Tg110 mice, which may facilitate conversion to classical BSE prions.

Two hypotheses for prion strain propagation in cross-species transmission experiments have been proposed: conformational selection favours a particular strain conformation out of a mixture of conformations in a scrapie isolate whilst mutation results in the conformational shift of one conformation into another [51]. Following on from the study in mice [17], it has been subsequently suggested that classical BSE properties that arise in atypical scrapie isolates transmitted to cattle may be due to conformational mutation in a new host [52]. It does not confirm that the atypical scrapie agent is the origin of the classical BSE epidemic and further transmission studies would be required to see whether classical BSE can be generated.

Would PMCA applied to brains from cattle exposed to TSE agents other than classical BSE and atypical scrapie also produce a classical BSE-like molecular phenotype? The PMCA product obtained in the thermostability test using a thermosensitive classical scrapie control showed a profile unlike classical BSE. Atypical BSE has been linked to the origin of classical BSE because of its conversion into classical BSE following serial passages in wild-type mice (L-type BSE [11]) and bovine transgenic mice (H-type BSE [53]). Although we have not tested PMCA products of atypical BSE isolates as part of this study, there is no evidence that PMCA products from atypical BSE convert into classical BSE, at least for H-type BSE using bovine brain as substrate [54]. In fact, we were unable to propagate H-type BSE using the same methodology (S Canoyra, A Marín-Moreno, JM Torres, unpublished observation).

The study results support the decision to maintain the current ban on animal meal in feedstuffs for ruminants, particularly as atypical scrapie occurs world-wide, and eradication is unlikely for a sporadic disease.

In summary, experimental inoculation of cattle with the atypical scrapie agent may produce clinical disease indistinguishable from classical BSE, which cannot be diagnosed by conventional diagnostic tests, but prions can be amplified by ultrasensitive tests in both clinically affected and clinically unremarkable cattle, which reveal classical BSE-like characteristics. Further studies are required to assess whether a BSE-like disease can be confirmed by conventional tests, which may initially include a second passage in cattle.

### Supplementary Information

**Additional file 1. Video file of steer 6/17, which is housed with a non-inoculated younger calf as pen mate, at 48 mpi.** The steer is slightly hesitant leaving its pen. Muscle tremor is evident when the steer is in the crush. When encouraged to move forward in the crush it kicks out. It also kicks out when the hind limb is touched with a stick, which is repeatable (positive stick test). There is no reaction to sudden light (negative flash test) when the animal’s head is restrained in the yoke, but ears move frequently, and a fine head tremor is visible, most noticeable when looking at the saliva dripping from its mouth. When released from the crush the steer leaps out rather than exiting calmly. Behavior and gait in the corridor with its pen mate appear normal. The steer has slight difficulty getting up characterized by rocking several times before rising on its hind legs (rising score 1).

## Data Availability

All data generated or analysed during this study are included in this published article and its Additional file.
